# Influence of Algae Age and Population on the Response to TiO_2_ Nanoparticles

**DOI:** 10.3390/ijerph15040585

**Published:** 2018-03-25

**Authors:** David M. Metzler, Ayca Erdem, Chin Pao Huang

**Affiliations:** 1 School of Arts and Sciences, Gwynedd Mercy University, Gwynedd Valley, PA 19437, USA; metzler.d@gmercyu.edu; 2 Department of Environmental Engineering, Akdeniz University, Antalya 07058, Turkey; 3 Department of Civil and Environmental Engineering, University of Delaware, Newark, DE 19711, USA; huang@udel.edu

**Keywords:** algae age, lipid peroxidation, chlorophyll, TiO_2_ nanoparticles

## Abstract

This work shows the influence of algae age (at the time of the exposure) and the initial algae population on the response of green algae *Raphidocelis subcapitata* to titanium dioxide nanoparticles (TiO_2_ NPs). The different algae age was obtained by changes in flow rate of continually stirred tank reactors prior to NP exposure. Increased algae age led to a decreased growth, variations in chlorophyll content, and an increased lipid peroxidation. Increased initial algae population (0.3−4.2 × 10^6^ cells/mL) at a constant NP concentration (100 mg/L) caused a decline in the growth of algae. With increased initial algae population, the lipid peroxidation and chlorophyll both initially decreased and then increased. Lipid peroxidation had 4× the amount of the control at high and low initial population but, at mid-ranged initial population, had approximately half the control value. Chlorophyll a results also showed a similar trend. These results indicate that the physiological state of the algae is important for the toxicological effect of TiO_2_ NPs. The condition of algae and exposure regime must be considered in detail when assessing the toxicological response of NPs to algae.

## 1. Introduction

Titanium dioxide (TiO_2_) nanoparticles (NPs) are extensively used in various industrial and consumer products [1,2,3,4]. As the production and usage of TiO_2_ increases, the release of NPs via spills, end use, and/or degradation of products into the environment also increases. The expected environmental loads of TiO_2_ NPs are 2−6 × 10^6^ tons [5], and the estimated worldwide production of TiO_2_ NPs is up to 10^5^ tons/year [6]. In addition to those, Keller et al. [7] estimated the total TiO_2_ NP release into air, water, soil, and landfill as 88,000 tons/year. As a result, the expected environmental concentrations of TiO_2_ NPs are higher than other metal oxide NPs due to their excessive production amounts and applications [7]. The predicted concentrations of nano-TiO_2_ in freshwater and in sediment are at ppb and ppm levels, respectively [8,9,10,11].

TiO_2_ generates reactive oxygen species (ROS) such as superoxide anions, hydrogen peroxide, and singlet oxygen, which can be harmful to organisms. The magnitude to which the ROS are generated depends on particle size, quantum yield, and light intensity. TiO_2_ has an activation wavelength between 376 and 413 nm in the UV range of the electromagnetic spectrum [12,13,14,15]. Studies have shown the effects of UV radiation [16,17,18,19], solar simulated light [20,21,22,23,24,25,26], and natural sunlight [27,28] with TiO_2_ NP on aquatic organisms in terms of mortality, reduced growth, and DNA deformation.

Green algae have been used as an indicator organism to study the toxicity of nanomaterials due to the ease of cultivation and performing lab-based assessments [29]. Standard procedures for toxicology tests are agreed upon by experts in toxicology, chemistry, and biology so that results are comparable. From these results, conclusions can be drawn about and between toxicants. Standard protocols for algae have been implemented around the world, including the Guideline for Testing of Chemicals, No. 201: Freshwater Alga and Cyanobacteria, Growth Inhibition Test [30], US EPA-821-R-02-013.2002: Short-Term Methods for Estimating the Chronic Toxicity of Effluents and Receiving Waters to Freshwater Organisms [31], and ISO 8692: Water Quality—Fresh Water Algal Growth Inhibition Test with Unicellular Green Algae [32]. These methods are implemented for legislative authorities to use as references and compare with studies that present data with similar initial algae density, light intensity, and organism age values. The results, although comparable, might not truly represent the natural environment. Cell populations and cell age are not constant as they are in a laboratory setting. Factors that are not constant in the natural environment include light intensity, algae density, and cellular age [33].

Limited studies have shown the effect of age on the toxicity of metals to various species. Hoang and Klaine [34] studied the toxicity of Cu, Zn, Se, and As on *Daphnia magna*. After dosing organisms at various ages with a 12 h pulse of one of the metals, biphasic toxicity with respect to mortality and reproduction was observed over 21 days of the experiment. *D. magna* mortality increased, while reproduction decreased as age increased. Rogevich et al. [35] showed the effect of age on Cu toxicity to the Florida apple snail (*Pomacea paludosa*). As the age of the organism increased at the time of exposure from 2 to 120 days, the 96 h LC_50_ (lethal concentration for 50% of the population) increased from 34.2 ± 4.4 to 182 ± 65.1 μg/L Cu. In the study by Lesser [36], changes in the maximum rate of photosynthesis (P_max_) was observed. As the experimental culture age increased from 0 to 22 days, the P_max_ decreased, while the shape of the curve remained nearly the same. The toxicity of Ni was most strongly correlated to that of the age of *Pimephales promelas* [37]. Stevenson et al. [38] showed the responses of *Chlamydomonas reinhardtii* from fast, slowing, and stationary growth phases to silver (Ag) NPs in terms of chlorophyll (*Chl*) measurements. Algae from fast growing phases showed more sensitivity to Ag NPs than did those from later phases. The *Chl* content of fast-growth-phase algae decreased from 0.3 (Day 1) to 0.01 μg/L (Day 3). The results from slow-growth-phase cultures showed a two-step decline during the exposure: (i) within the first three days, *Chl* content decreased from approximately 100 to 30 μg/L following a 10 μg/L of recovery; (ii) in the last four days, *Chl* content decreased from approximately 40 to 20 μg/L.

The age of organisms can affect the toxicity of stressors. The interaction between the stressor and organism is an important factor, known as body burden. Body burden can be defined as the amount of chemical that is present in a body at a given time, usually given in mass of chemical per mass of organism. This term is more directly related to the toxicological effect than exposure concentration due to the extent of bioavailability of a toxicant. Two methods can be used to vary the body burden: (1) varying the population density while the toxicant concentration is kept constant or (2) varying the toxicant concentration while the populations are held constant. Moreno-Garrido et al. [39] demonstrated how varying the initial algal population density affects the EC_50_ (half maximal effect concentration) values of the toxicant—in this case, Cu. The authors refer to the exposure per algae cell as the “toxic cellular quota.” The authors asserted that there was a linear relationship between the log Cu cellular quota and the % growth inhibition. Table 1 summarizes the results of the studies employing different initial algae populations, test media, ionic strength, and particle sizes. The results were depicted in EC_50_ (mg/L). Most of the studies used an algal medium as a test solution, and, even though the same protocols [30,31] were used, the initial algae population varied between 10^4^ and 3.5 × 10^6^ cell/mL, and the ionic strength of the test solutions varied from 0.5 to 8 mM. As a result of using different test media with different ionic strengths and other conditions (not depicted in Table 1), the particle sizes and EC_50_ values were not comparable.

Since the physiological state and the age of algae are important factors determining their response to any toxicant, different initial cell densities and cell ages were applied in our study to address two questions: (i) whether various initial cell densities have an important role in ecotoxicity of TiO_2_ NPs, and (ii) how the physiology of algae affects the ecotoxicity of TiO_2_. 

## 2. Materials and Methods 

### 2.1. Algae

Algae specimens of *Raphidocelis subcapitata* (formerly *Pseudokirchneriella subcapitata* and *Selenastrum capricornutum*) were purchased from Aquatic Biosystems (Fort Collins, CO, USA). The algal inoculums were stored at 4 °C in the dark for no longer than 6 months. It was cultured in algal medium using a continually stirred tank reactor (CSTR) according to Metzler [43]. Algal growth medium (0.642 M) was prepared with macronutrients (MgCl_2_·6H_2_O: 6.08 g, CaCl_2_·2H_2_O: 2.20 g, NaNO_3_: 12.75 g, MgSO_4_·7H_2_O: 7.35 g, K_2_HPO_4_: 0.522 g, NaHCO_3_: 7.50 g) and micronutrients (H_3_BO_3_: 92.8 mg, MnCl_2_·4H_2_O: 208.0 mg, ZnCl_2_: 1.64 mg, FeCl_3_·6H_2_O: 79.9 mg, CoCl_2_·6H_2_O: 0.714 mg, Na_2_MoO_4_·2H_2_O: 3.63 mg, CuCl_2_·2H_2_O: 0.006 mg, Na_2_EDTA·2H_2_O: 150.0 mg, Na_2_SeO_4_: 1.196 mg) dissolved in 500 mL milli-Q water [31].

The algae solutions used in zeta potential measurements were filtered using glass fiber filter paper (Whatman Inc., Florham Park, NJ, USA). The filter paper was soaked in an Erlenmeyer flask containing a 0.65 mM NaCl solution. The pH of the algal suspensions was adjusted to 3.0, 4.5, 5.6, 7.0, and 9.2 with varying amounts of HCl, NaHCO_3_, and Na_2_CO_3_, and the zeta potential of the algal solutions was finally measured using a Malvern Nano-ZS (Westborough, MA, USA).

In order to test the effect of the initial population density, the CSTRs had a 3-day hydraulic retention time (*HRT*). Initial cell densities were between 0.31 and 4.19 × 10^6^ cells/mL in the sample. When the algal population age was tested, the *HRT* of the CSTRs were varied between 2 and 25 days, with a cell density of 9.77 ± 4.87 × 10^5^ cells/mL in sample. Prior to the addition of the algae to the test beakers, the absorbance, cell density, and *Chl a* were measured using a HACH spectrophotometer [38]. Experiments were run in triplicate.

### 2.2. Nanoparticles

TiO_2_ (P25) was purchased from Degussa Corp. (Parsippany, NJ, USA). The surface area was measured with N_2_-sorption with a Quantachrome model Nova 2000. TiO_2_ NP particle size was calculated using BET surface area results, assuming that TiO_2_ NPs were spherical. The particle size and the zeta potential of TiO_2_ were measured using a Malvern Nano-ZS Particle and ZetaSizer (Westborough, MA, USA). Dynamic light scattering (DLS) was performed to calculate the particle sizes.

Stock suspensions of either 2 or 4 g/L were made in algal growth media [6] without any prior treatment to the nanoparticles. The suspensions were autoclaved for 20 min at 120 °C at least one day prior to use, and stored at 21 ± 1 °C. Prior to the exposure experiments, sonication was applied using an ultrasonic homogenizer (600 W, 20 kHz, Cole-Parmer, Chicago, IL, USA) equipped with a titanium probe transducer (Model CV 17, Cole Parmer) for 5 min at an energy intensity of 24 W.

### 2.3. Test Chambers

**Figure 1:**
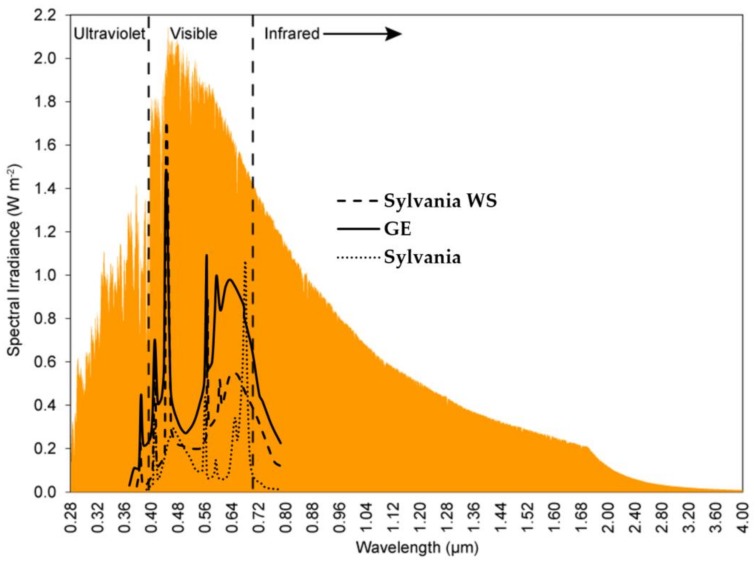
Light spectrum of solar light (orange colored area) [44] and light sources used in the study (Sylvania WS (---): F12T20/Gro/AQ/WS, GE (^___^): GE PL/AQ F12T20, and Sylvania (^….^): Gro-lux F12T20/Gro/AQ).

Three test chambers with dimensions of 80 × 60 × 70 cm (H × W × L) were covered in black cloth. An orbital platform shaker holding eight, 1 L plastic beakers was used to conduct the exposure experiments. One bank consisting of six lights (model GE PL/AQ F12T20, General Electric, Fairfield, CT; Sylvania Gro-lux F12T20/Gro/AQ and F12T20/Gro/AQ/WS, Rutherford, NJ, USA) was suspended approximately 42 cm from the bottom of the test beakers. The light intensity at the top of the beaker was 1632 ± 409 lux. The light sources emitted light at a spectrum mimicking that of solar light (Figure 1). A centrifugal mini-blowers and a muffin fan, one on each side of the chamber, removed excess heat generated by the lights. The shaker was set to mix at 150 rpm throughout all experiments.

### 2.4. Exposure Regime

Aliquots of particles were added to 1 L plastic beakers to make final concentrations of 100 or 1000 mg/L. Algal growth media was then added to the beakers (≤100 mL) in various volumes depending on the experimental setup listed below. The samples were then sonicated for 2 min at 48 W (8% of 600 W) with a 9 mm probe using a Cole Parmer Ultrasonic Homogenizer Series 4710 (Cole Parmer, Chicago, IL, USA). The samples were then allowed to equilibrate for 1 h, after which the % transmission was measured on a Hewlett-Packard 8542A UV-vis (Fall River, DE, USA), and the spectrum was recorded. Once the algae were added, the final volume was 200 mL. Three replicates were run on separate occasions.

### 2.5. Measurements

The pH and the temperatures of the growth chambers were measured each day to ensure stability. At the end of the 4-day exposure, the remaining sample volume was determined. Forty milliliters of sample was collected to analyze *Chl a*, and 12 mL was collected to analyze cell density and lipid peroxidation. The samples were stored at 4 °C in the dark until analyzed. Weber et al. [45] reported that *Chl* content would remain stable for a maximum of seven days.

Cell densities were measured by direct cell counts. Sample (1 mL) was diluted in 0.5 moles/L lauryl sulfate (1 mL) and vortexed for 2 min. A 100 μL of each sample was counted 4 times using a hemocytometer, and Olympus AX70 microscope. Cell densities were converted into total cell population by multiplying the cell counts by the volume of sample remaining in the 1 L test beakers at the end of the test duration. The total cell populations were then corrected for the initial population and normalized based on the control, using the following equation:

(1) $$R_{s}=\;\frac{{\left(\rho \;\times \;V\right)}_{t}}{{\left(\rho \;\times \;V\right)}_{c}}$$

where *Rs* is the normalized specific growth (NSG), *ρ* is the cell density (cells/mL), *V* is the volume of sample at the end of the 4-day exposure period (mL), *t* is the sample exposed to NPs, and *c* is the control sample with no treatment.

To analyze *Chl a* content, 40 mL of sample were concentrated to between 0.5 and 3 mL *Chl* [39]. The concentrated samples were brought to 3 mL with Mg–acetone. The samples were homogenized for 1 min at 2000 rpm and were then steeped at 4 °C in the dark for 2 h. After the steep time, the samples were centrifuged for 30 min at 664 g. The optical density of the supernatant was measured at 750 and 664 nm with a UV-vis spectrophotometer (HP 8525A). HCl (0.1 mL of 0.1 N) was added to the samples. After 90 s, the optical density was re-measured at 750 and 665 nm. The concentration of *Chl a* ([*Chla*] in mg-*Chla*/m^3^)) was calculated using the following equation:

(2) $$\left[Chla\right]=\;\frac{26.7\;\times \left(A664-A665\right)\times V_{1}}{V_{2}\times l}$$

where *A*664 is the absorbance at 664 nm before adding acid, *A*665 is the absorbance at 665 nm after adding acid, *V*_1_ is the volume of the extract (L), *V*_2_ is the volume of sample (m^3^), and *l* is the width of the cuvette (cm). The specific *Chl a* was calculated by the following expression:

(3) $$y=\;\frac{\left[Chla\right]}{\rho \;\times \;10^{6}}\;$$

where *y* is the specific *Chl a* (mg *Chla*/cell), [*Chla*] is the concentration of *Chl a* (mg *Chla*/m^3^), and *ρ* is the cell density (cells/mL). The specific *Chl a*, *y*, was then normalized with respect to the control condition according to the following equation:

(4) $$Y=\;\frac{y}{y_{c}}$$

where *Y* is the normalized specific *Chl a* (pg *Chla*/cell), *y* is the specific *Chl a* of samples (mg *Chla*/cell), and *y_c_* is the specific *Chl a* of the control (mg *Chla*/cell).

Lipid peroxidation method was used following Maness’ malondialdehyde (MDA) method [46]. A 1 mL sample was added to 2 mL of 10% trichloroacetic acid (TCA). The mixture was centrifuged for 45 min at 11,000 *g*. The supernatant was added to 3 mL of 6.7 g/L 2-thiobarbituric acid (TBA). The samples were heated in boiling water for 10 min. After cooling, the samples were measured on a UV-vis spectrophotometer at 532 and 600 nm wavelengths. The absorbance at 532 nm was corrected for the background at 600 nm by subtracting the measurement at 600 nm from those at 532 nm. Calibration standards were made from 1,1,3,3-tetramethoxypropane. Normalized specific lipid peroxidation (*Z*) was calculated by the following equations:

(5) $$z=\;\frac{\left[MDA\right]}{\rho \;\times \;10^{3}}$$

where *z* is the pmol *MDA*/cell, [*MDA*] is the concentration of *MDA*-TBA acid complex measured (μM), and *ρ* is the cell density (cells/mL). The *z* value was then normalized against the control according to the following equation:

(6) $$Z=\;\frac{z}{z_{c}}$$

where *Z* is the specific lipid peroxidation of the sample (pmol *MDA*/cell), and *z_c_* is the specific lipid peroxidation of the control (pmol *MDA*/cell).

## 3. Results and Discussions

### 3.1. Nanoparticle Characterization

Since the surface chemistry of the NPs and the solution composition and chemistry play an important role in the process of aggregation/agglomeration of NPs [47,48], we obtained the surface area, size, and zeta potential values of TiO_2_ NPs in algal media. The surface area of the TiO_2_ NPs before exposure was measured with N_2_-sorption with a Quantachrome model Nova 2000 and found to be 44.0 ± 2.9 m^2^/g. The particle sizes before (*t* = 0) and after (*t* = 72 h) the exposure measured with the DLS method were 35.1 ± 2.1 nm and 294 ± 28 nm, respectively.

**Figure 2:**
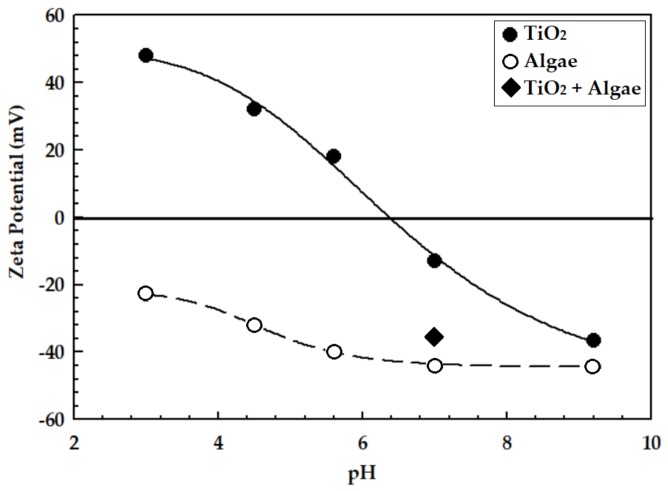
Zeta potential values of algae and TiO_2_ NPs as a function of pH.

The zeta potential values of algae, TiO_2_ NPs, and algae+TiO_2_ NPs are shown in Figure 2. The zero point charge (pHzpc) of TiO_2_ NPs was 6.4 and the same value was also reported by Lin et al. [49], where other studies showed pHzpc as 6.25 [50] and 6.3 [51]. The NPs and algae were negatively charged during the experiments. The zeta potential of the algae+TiO_2_ NP solution was also measured and found to be −33 mV at pH 7.3 ± 0.5. Similarly, it has been reported that with different algae per TiO_2_ NP ratios, the zeta potential measurements were between −32 and −36 mV when the pH of the solution was around 7.2 [49]. Guzman et al. [52] noted that the pH of the solution increases the aggregation/agglomeration of the NPs when it approaches the pHzpc. Othman et al. [53] showed an enhanced stability of NPs with higher zeta potential magnitude. Ozkaleli and Erdem [26] reported a moderate stability of TiO_2_ NPs in a very soft synthetic surface water samples with an ionic strength of 0.5 mM. In our results, a moderate stability was also achieved in algae+TiO_2_ NP solutions.

### 3.2. Initial Algal Population

Laboratory protocols define specific parameters to test the effects of certain chemicals on indicator organisms; however, the natural environment does not always fall under these defined conditions. Therefore, a series of experiments were conducted to determine how population age and density affect the response to TiO_2_ NPs.

**Figure 3:**
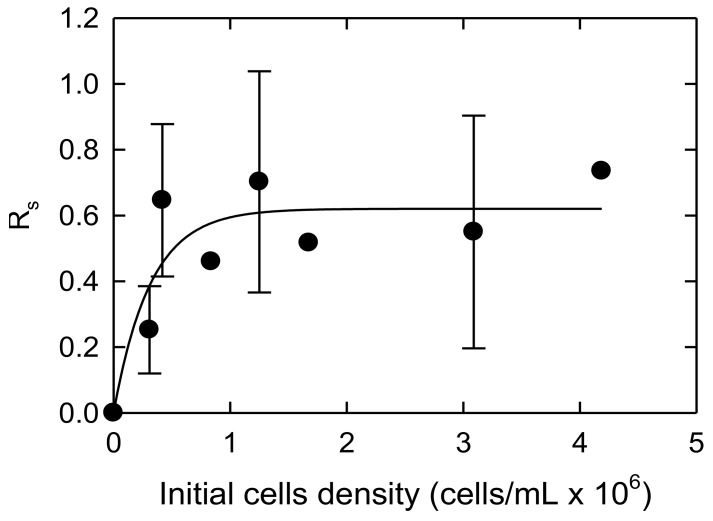
Normalized specific growth plotted against the initial cell density. The line is fitted using the empirical Equation (7).

**Figure 4:**
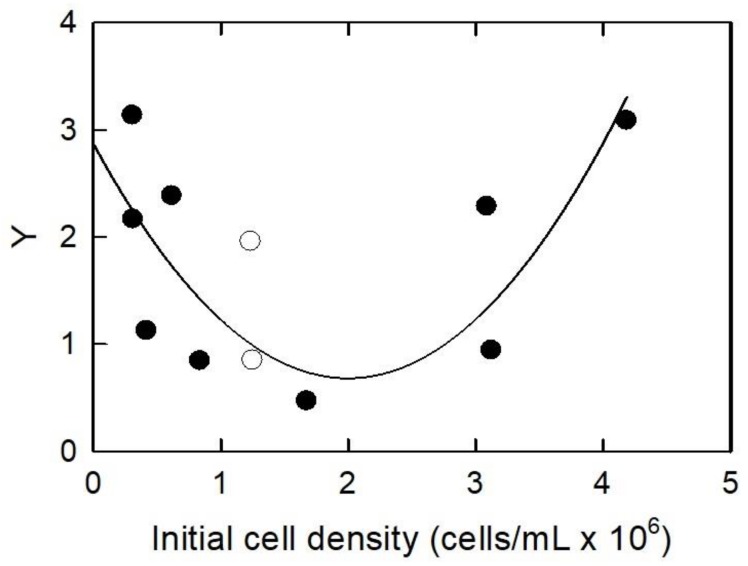
Normalized specific chlorophyll a plotted against the initial cell density. Closed symbols are data points. Open symbols are data that were considered outliers. Values of outliers are 11× plotted value. Line is fitted using the empirical Equation (8).

**Figure 5:**
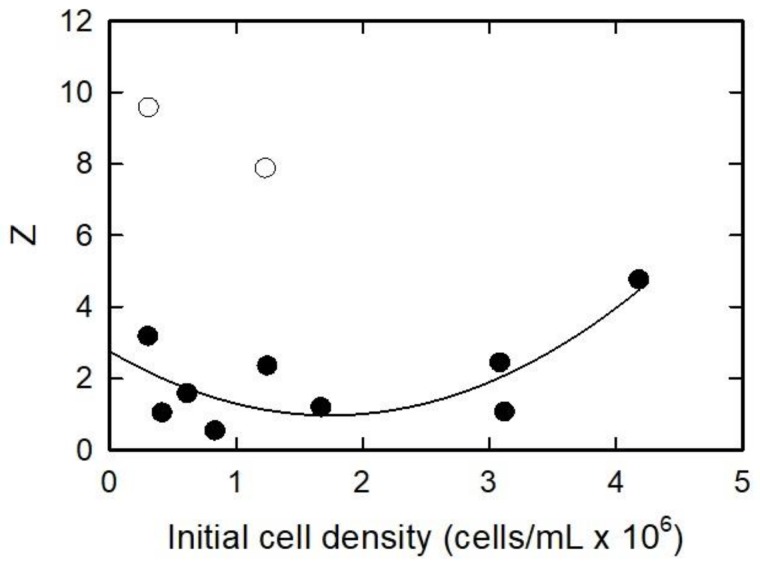
Normalized specific lipid oxidation plotted against the initial cell density. Closed symbols are fitted data points. Open symbols are data that were considered outliers and not included in the fitting. Line is fitted using the empirical Equation (9).

The effect of body burden or TiO_2_ cellular quota was investigated by varying the initial cell density, while holding the concentration of 35.1 ± 2.1 nm TiO_2_ NPs constant (100 mg/L), and the results are shown in Figure 3, Figure 4 and Figure 5. The temperature for those experiments was 22.9 ± 1.2 °C. The average pH of the samples was 7.3 ± 0.5.

Figure 3 shows the effect of different initial cell population on NSG. As the initial cell population increased from 0.5 × 10^6^ cells/mL to 0.75 × 10^6^ cells/mL, an initial increase in the NSG of 0.1–0.5 was observed. However, with an initial cell population greater than 0.75 × 10^6^ cells/mL, the NSG showed a constant value of approximately 0.6. The data was modeled with the following equation as follows:

(7) $$R_{s}=\;R_{smax}\times \left[\frac{\beta \times \rho_{i}}{1+\left(\beta \times \rho_{i}\right)}\right]$$

where *R_s_* is the normalized cell growth, *R_smax_* is the maximum normalized cell growth, [*ρ_i_*] is the initial cell density, and *β* is a curve shape factor. The data was fitted in an Excel solver, where *R_smax_* = 0.685, *β* = 4.673, and the root mean squared error (RSME) = 0.498. As the number of initial cells increased, the effect of the stress from the NPs decreased. This stress from body burden reached a steady state of around 1 × 10^6^ cell/mL.

Figure 4 represents the normalized specific *Chl a* (*Y*) for varying initial cell concentrations at 100 mg/L TiO_2_ NPs. As cell population increased from approximately 0.5 × 10^6^ to 1 × 10^6^ cell/mL, the *Y* decreased from approximately 3.1 × 10^6^ to 0.5 × 10^6^, of the control. From an initial cell population of approximately 1.8 × 10^6^ to 3.1 × 10^6^, there was no change in *Y*. As the initial cell population increased from 3.1 × 10^6^ to greater than 4 × 10^6^ cells/mL, *Y* increased from approximately 1 × 10^6^ to 3.1 × 10^6^, of the control. Two outlier data points were excluded from the set. The outliers are represented by open circle symbols in Figure 3. To fit the data on the graph, the outlier values were divided using Equation (8). The data was fitted in SigmaPlot ver. 9.01 with empirical second-order polynomial Equation (8).

(8) $$Y=\;Y_{0}+a\left(\rho_{i}\right)+b{\left(\rho_{i}\right)}^{2}$$

Y_0_ is the minimum value of the normalized specific *Chl a*, [*ρ_i_*] is the initial cell density (cells/mL), and *a* and *b* are the fitting parameters (r^2^ = 0.547). At a steady concentration of NPs, the number of nanoparticles on each algae cell increased as the initial cell density decreased. These results agree with results from Wu et al. [54], who found that *Chl a* content increased under low light conditions, and high light conditions produced more *Chl a* than an intermediate photon flux. The amount of light reaching the algae cells in this experiment would vary with the concentration of NPs per algae cell. This reinforces the idea of a shading effect, where less light reaches the algae cells.

Figure 5 shows the normalized lipid peroxidation (*Z*) of different initial cell populations. As the initial cell density increased from 0.5 × 10^6^ to about 2 × 10^6^ cells/mL, the *Z* decreased from approximately 2 to 1. As cell density increased greater than 2 × 10^6^ cells/mL, the *Z* increased to about 5. The data was fitted in SigmaPlot ver. 9.01 with an empirical second-order polynomial:

(9) $$Z=\;Z_{0}+c\left(\rho_{i}\right)+d{\left(\rho_{i}\right)}^{2}$$

*Z*_0_ is the normalized specific lipid peroxidation minimum value, (*ρ_i_*) is the initial cell density (cells/mL), and *c* and *d* are the fitting parameters. The data fit the model reasonably well with an r^2^ of 0.598. During photosynthesis, reactive oxygen species (ROS) are generated. Under a moderate stress this can lead to the chloroplast releasing an excess amounts of ROS, which the cell cannot regulate [55]. From these results, the increased *Z*_0_ at low initial cell densities could be attributed to an excess of internal ROS species from the increased *Chl a* on a per cell basis while the cells are under stress. The algae cells are considered under “moderate stress” as defined by Dietz et al. [55] under the conditions of the experiment. As the initial cell density increases the algae cell are less stressed, on a per cell basis. The algae cells produce less *Chl a* per cell, which will result in less internal ROS and the *Z* value will decrease. At the initial cell densities above 2 × 10^6^ cells/mL, the *Z* increase is attributed to the interaction of the ROS generated from the TiO_2_ NPs.

### 3.3. Algae at Various Hydraulic Residence Times 

The toxicological response of organisms has been linked to the age at which the organism was exposed. Muyseen and Janssen [56] observed that *D. magna* had different responses to Zn and Cu, where the juveniles were more or equally as sensitive. The increased sensitivity was attributed to larger area/volume ratios, which led to a size-dependent uptake and higher metabolic rates of younger organisms. Similar findings were observed by Yu and Wang [57], Hutchinson et al. [58], and Stuhlbacher et al. [59]. The response to a stressor can be affected by an organism’s physiological state. Bacteria and algae grown in a CSTR can be cultured to maintain the organism in a given physiological state, where the time is the flow rate of the CSTR.

**Figure 6:**
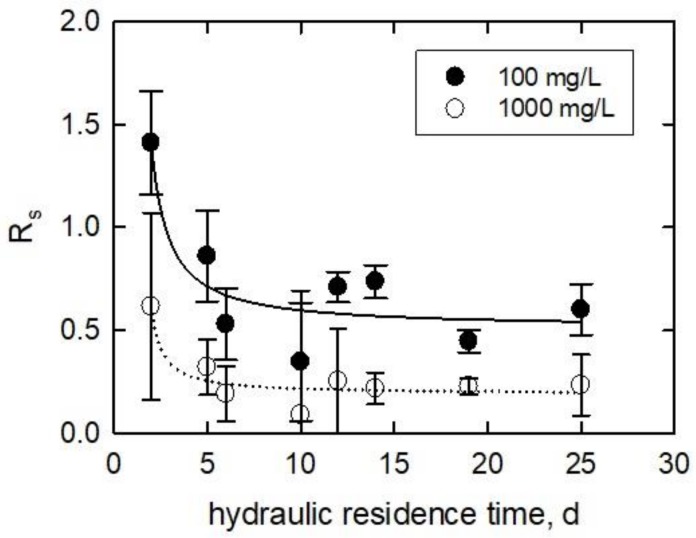
Normalized specific cell growth plotted against the hydraulic residence time of the continually stirred tank reactors in which the algae inoculums were cultured. Lines are fitted using Equation (10).

**Figure 7:**
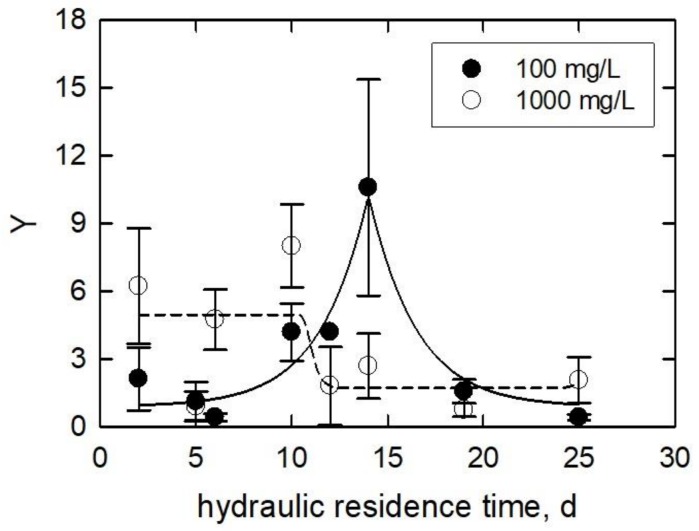
Normalized specific chlorophyll a plotted against the hydraulic residence time of the continually stirred tank reactors in which the algae inoculums were cultured. Lines are fitted using Equation (11).

**Figure 8:**
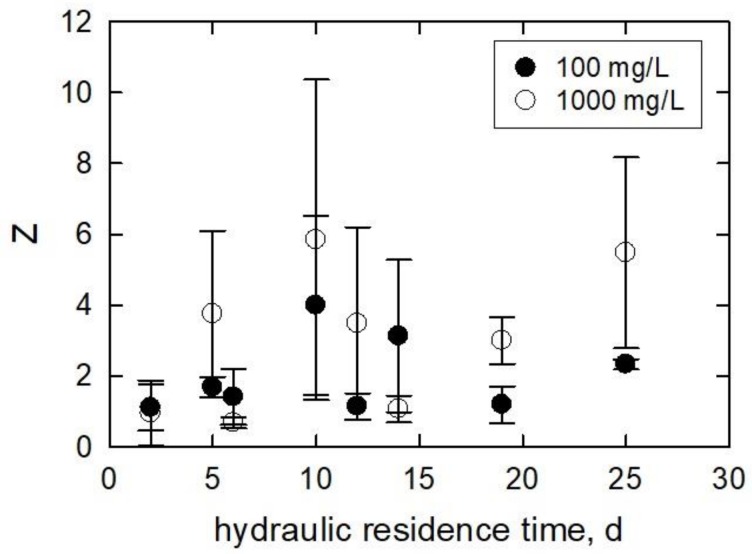
Normalized specific lipid peroxidation (*Z*) plotted against the hydraulic residence time of the continually stirred tank reactors in which the algae inoculums were cultured.

**Figure 9:**
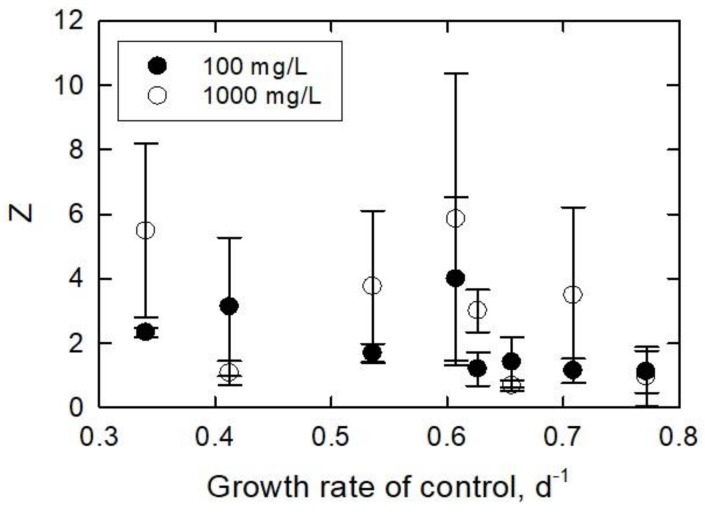
Normalized specific lipid peroxidation plotted against the measured growth rates of the control samples.

The growth phase can be set by varying the flow rate in a CSTR. Therefore, flow rates were maintained between 5 and 62 mL/h, which correspond to a 25 and 2 day *HRT* (*d*). Figure 6, Figure 7, Figure 8 and Figure 9 show the results of culturing the algae at various *HRT* values, and the response (R_s_, *Y*, and *Z*) to two concentrations of 35.1 ± 2.1 nm TiO_2_. The temperature for this experiment was 23.0 ± 1.2 °C. The average pH of the samples was 7.5 ± 1.0.

Figure 6 shows a plot of the *R_s_* against the *HRT*. As the *HRT* for the 100 mg/L treatment (from 2 to 10 *HRT*) increased, the *R_s_* deceased for both 100 and 1000 mg/L treatments. The response is biphasic. The *R_s_* decreases from 1.4 to 0.35 as the *HRT* increased to 10 d. At *HRT* > 10, the values for *R_s_* are between 0.35 and 0.74, with a slope of near zero. For the 1000 mg/L treatment, the *R_s_* decreased from 0.62 to 0.09 from 2 to 10 *HRT*, respectively. Moreover at *HRT* > 10, the *Rs* for the 1000 mg/L treatment remains relatively constant at 0.23. The data was fitted a modified three-parameter exponential decay equation from SigmaPlot version 9.01. The equation used is as follows:

(10) $$R_{s}=f\times e\left(\frac{g}{HRT+h}\right)$$

where *HRT* is the hydraulic retention time (*d*), and *f*, *g*, and h are fitted constants. *R*-squared values were 0.785 and 0.842 for the 100 and 1000 mg/L TiO_2_ treatments, respectively.

Figure 7 is the *Y* plotted as a function of *HRT*. In the 100 mg/L treatment, the *Y* increased from 2 to 10 as the *HRT* increased from 2 to 16 days. From 16 to 25 days, the *Y* decreased from 10 to 1. The data was empirically fitted to a modified five-parameter Gaussian equation from SigmaPlot version 9.01. The equation is as follows:

(11) $$Y=Y_{0}+i\times e\left(-0.5k\frac{HRT-HRT_{0}}{j}\right)$$

*Y*_0_ is the minimum value of the normalized specific *Chl a*, *HRT* (d) and *HRT*_0_ (d) are the hydraulic residence time at which algae were cultured and the minimum value for *HRT*, respectively, and *i*, *j*, and *k* are fitted constants.

For the 100 mg/L treatment, the r^2^ is 0.93. From this equation, the maximum *Chl*/cell was produced at an *HRT* of 14 days. The equation does not fit the data for the 1000 mg/L treatment as well as the 100 mg/L, with an r^2^ of 0.40 from Equation (10). At a small *HRT* (2–10), the 1000 mg/L treatment increased the *Y* by an average of 6× over the control. The *Y* then decreased from between *HRT* values of 12 and 25 days (100 mg/L TiO_2_) to between *HRT* values of 3 and 1 (1000 mg/L TiO_2_). 

Kulandaivelu and Senger [60] showed an 85–98% decrease in photosynthetic activity from *Scenedesmus obliquus,* heterotrophically grown algae for 10–30 days, respectively. In another study, a decline in photosynthesis rate was reported when algal culture gradually reached the stationary growth phase [61]. Cheregi et al. [62] studied the presence of state transitions in different culturing ages of *Guillardia theta* in terms of *Chl a* and *c* measurement. The algae were grown for 1–2 days (early logarithmic phase), 2–6 days (logarithmic phase), 6–9 days (early stationary phase), and 9–13 days (late stationary phase). Their result showed that the amount of *Chl a* and *c* declined from 1.34 pg/cell (2-day culture) to 0.750 pg/cell (13-day culture). Oukarroum [63] evaluated the photosynthetic activity of *C. vulgaris* and *S. obliquus* from different growth phases using in vivo *Chl a* fluorescence transient measurement. Algae age and species significantly affected the photosynthetic activity that, after 20 days, *Chl a* content declined by 84% in *C. vulgaris* and 89% in *S. obliquus* compared to the values from Day 3. Figure 8 is the normalized specific lipid peroxidation (*Z*) plotted as a function of *HRT*. In most cases, *Z* results were greater for the 1000 mg/L treatment than the 100 mg/L treatment. There is no clear trend in the data. The maximum *Z* of 4.0 ± 2.5 occurred in the 100 mg/L treatment at an *HRT* of 10 days, while the minimum of 1.1 ± 0.6 occurred in this treatment at an *HRT* of 2 days. The maximum and minimum *Z* values for the 1000 mg/L treatment were 5.8 ± 4.5 and 0.68 ± 0.16 at *HRT* values of 10 and 6 days, respectively. In all but in three cases, the 1000 mg/L treatment generated more lipid peroxidation. Two of those cases had similar values with relatively small standard deviations. Comparison between the two treatments shows that the *Z* trends are the same, i.e., the slopes increase or decrease together. When the *Z* was plotted against the growth rate (calculated by Equation (12), day^−1^) of the control samples, a trend emerged (Figure 9).

(12) $$k=\frac{\ln\left(\rho_{i}\right)-\;\ln\left(\rho_{f}\right)}{\Delta t}$$

*k* is the growth rate of the controls under experimental conditions (d^−1^) of each *HRT*, *ρ_i_* is the initial cell density of the controls of each *HRT* (cells/mL), ρ_t_ is the final cell density of the controls of each *HRT* at the end of the experimental duration (cell/mL), and Δt is the duration of the experiment (d). At low growth rates (0.35–0.6), the lipid peroxidation per cell was about 6× and 3× greater than the control for the 1000 and 100 mg/L treatments, respectively. Only in one case (0.41 day^−1^) was the *Y* lower in the 1000 mg/L treatment than in the 100 mg/L treatment. At growth rates >0.6, the *Y* had values similar to those of the control samples in the 100 mg/L treatment. The 1000 mg/L treatment caused the *Y* to decrease to control values as well; however, one sample produced higher *Y* at 4× the control at 0.72 day^−1^.

It is a well-known phenomenon that TiO_2_ NPs can generate reactive oxygen species (ROS) and hydroxyl radicals (OH·) under UV light. As being strong oxidants, ROS can decompose organic materials (i.e., cell surfaces) [64,65]. ROS cause oxidative stress, which leads to lipid peroxidation and toxicity to organisms [26,46,66,67,68,69]. In our previous studies [20,21], we have shown the growth inhibition of green algae, *R. subcapitata*, exposed to TiO_2_ NPs under fluorescence light irradiation. Lee and An [18] have shown the inhibition of *R. subcapitata* growth under visible light, UVA, and UVB irradiation conditions.

Kadukova et al. [70] employed algae *Parachlorella kessleri* from different culturing weeks (1–4 weeks) to produce Ag NPs. UV-visible spectral analysis was performed, and maximum peaks were obtained from one-week cultures. Within 14 days, culture age (1-4 weeks) and the Ag NP formation showed a linear relationship. After 15 days, the Ag NP production rate decreased in 1, 3 and 4 week cultures. Adeleye et al. [71] evaluated the effect of sulfide/silica-modified nZVI (FeSSi) on *Chlamydomonas reinhardtii* algae cultured for 1, 2, or 11 days. Regardless of the exposed FeSSi doses (1.8 and 18 mg/L), one-day cultures were more sensitive than 11-day cultures.

## 4. Conclusions

This research was undertaken to determine how population density affects the toxicity of 35.1 ± 2.1 nm TiO_2_ and how algae physiology affects the toxicity of TiO_2_. 

First, varying the initial algae cell concentration resulted in two reactions to the same mass of NPs. At high body burdens, there was an increase in *Z* and *Y*, while *R_s_* was depressed. *Z* results indicate that direct contact between NPs and algae increased lipid peroxidation due to the loading. ROS reaction rates are relatively fast in aqueous systems. Therefore, direct transfer of the ROS to surface lipids or carbohydrates will disrupt the cell wall/membrane functions.

Hydraulic retention varies the age of algae. The age of algae affects many physiological parameters. This includes the lipid content and growth rates of the algae. The toxicity of nano-TiO_2_ is age-dependent in the studied system. The toxicity/stress of NPs may be diluted on a per cell basis in fast growing samples. Thus, the physio-kinetics of growth and nanoparticle interaction is important. Additionally, age affects the composition of the cultures as well. ROS were more likely to affect the higher lipid content cells. This can be important in the environment, as time of year is affects the algae response to NPs.

The exposure regime must be taken into consideration when interpreting the toxicity of photocatalytic NPs with a 35.1 ± 2.1 nm TiO_2_ diameter. Additional NP sizes should be investigated for similar findings.

## Figures and Tables

**Table 1:** Data on ecotoxic effects of TiO_2_ nanoparticles on *Raphidocelis subcapitata.*

Initial Algae Population (Cell/mL)	Test Media Type	Calculated Ionic Strength (mM)	Particle Size (nm)	Half Maximal Effect Concentration (EC_50_, mg/L)	Reference No
10^4^	Algal medium	1.5	21	2.53	[18]
1.4 ± 0.9 × 10^6^	Algal medium	640	42	25.5	[21]
2.6 ± 1.1 × 10^5^	Synthetic freshwater solutions	0.5	212 ± 19	4.16 ± 0.05	[26]
		1	287 ± 25	3.58 ± 0.16	
		2	546 ± 71	9.32 ± 0.11	
		8	1428 ± 202	12.14 ± 0.09	
10^5^	Algal medium	640	140	87	[40]
10^4^	Algal medium	1.5	~30	71.1	[41]
3.5 × 10^6^	Algal medium	640	25–70	5.38	[42]
3 × 10^6^	Algal medium	620	140 and 380	87 and 61	[43]
